# Draft Genome Sequence of *Streptomyces* sp. B9173, a Producer of Indole Diketopiperazine Maremycins

**DOI:** 10.1128/genomeA.00447-17

**Published:** 2017-06-01

**Authors:** Fang Luo, Yi Zou, Tingting Huang, Shuangjun Lin

**Affiliations:** State Key Laboratory of Microbial Metabolism, Joint International Laboratory on Metabolic & Development Science, School of Life Sciences & Biotechnology, Shanghai Jiao Tong University, Shanghai, China

## Abstract

*Streptomyces* sp. B9173 is a producer of maremycins, a group of naturally occurring 2,5-diketopiperazines. Here, we report the draft genome sequence of *Streptomyces* sp. B9173, which comprises ~8.77 Mb, with a G+C content of 71.8%.

Actinomycetes are recognized as the richest source of variety bioactive compounds, among which members of the genus *Streptomyces* are the most prolific producers of secondary metabolites (1). The marine microorganism *Streptomyces* sp. B9173 was isolated from the sediment on the Pacific coast in Chile. *Streptomyces* sp. B9173 has been identified as *Streptomyces rishiriensis* based on the 16S rRNA gene sequence and phylogenetic analysis. Additionally, it has been confirmed to produce plenty of secondary metabolites, including the diketopiperazine derivatives maremycins (2, 3).

To obtain comprehensive information about these putative pathways and explore the potential to produce other novel products, the draft genome sequence was determined by a shotgun sequencing using 454 pyrosequencing technology on a GS FLX Titanium platform. A total of 485,128 reads were generated and assembled using the ABySS software (version 1.2.3). The final assembly consists of 133 contigs (*N*_50_, 135,224 bp), with an average size of 65,968 bp. The total assembled size was 8,773,824 bp, with a G+C content of 71.8%. Annotation was performed using the NCBI Prokaryotic Genome Annotation Pipeline (PGAP) (4) that utilizes GeneMarkS+ (5), BLASTn (6), and tRNAscan-SE (7), yielding a total of 7,695 predicted protein-coding sequences (CDSs), 74 tRNA genes, and 3 rRNA operons.

To identify secondary metabolite clusters, the draft genome sequence was analyzed by antiSMASH version 4.0 (8). This genome contains at least 31 biosynthetic gene clusters (BCGs), including 4 nonribosomal peptide synthetase (NRPS) gene clusters, 1 type I polyketide synthase (PKS), 1 type II PKS, 1 type III PKS, and 3 hybrid NRPS-PKS clusters. A hybrid NRPS-PKS gene cluster for maremycin biosynthesis was present in contig00017 (9, 10), which contains a typical *marG-H-I* cassette responsible for the β-methyl tryptophan moiety biosynthesis. The *marG-H-I* cassette homologs exist within the streptonigrin gene cluster in *Streptomyces flocculus* CGMCC 4.1223 (11) and the FR-90452 gene cluster in *Streptomyces* sp. TP-A0890 (12), which reveals a conserved β-methyl tryptophan biosynthetic machinery. A hybrid typeIII PKS-terpene gene cluster in contig27 and contig49 was predicted for the biosynthesis of flaviogeranin. An NRPS gene cluster was found in contig00034 showed 83% similarity to a paenibactin biosynthetic gene cluster in *Paenibacillus elgii* B69 (13). A hybrid NRPS-PKS gene cluster in contig117 shared 86% sequence similarity to the antimycin biosynthetic gene cluster in *Streptomyces* S4 (14). An NRPS located in contig00038 was predicted to synthesize a scabichelin type siderophore which was identical to the corresponding gene cluster in the plant pathogen *Streptomyces scabies* 87.22 (15). A type II PKS gene cluster responsible for chromomycin biosynthesis was presented in contig55 (3). The remaining gene clusters, except for those stated above, display no significant similarities to the gene clusters whose products are characterized. The prediction of other types of biosynthetic gene clusters reported here will accomplish the discovery of uncharacterized secondary metabolites.

## Accession number(s). 

This draft genome sequence of *Streptomyces* sp. B9173 has been deposited in the DDBJ/ENA/GenBank under the accession no. NAVC00000000. The version described in this paper is the first version, NAVC01000000.

## References

[1] Bérdy J 2005 Bioactive microbial metabolites. J Antibiot 58:1–26. doi:10.1038/ja.2005.1. 15813176

[2] Balk-Bindseil W, Helmke E, Weyland H, Laatsch H 1995 Marine bacteria, VIII. Maremycin A and B, new diketopiperazines from a marine *Streptomyces* sp. Liebigs Ann 1995:1291–1294. doi:10.1002/jlac.1995199507171.

[3] Xu X, Fang Q, Liang Z, Lin S 2015 Chromomycin A3 and its analogues from the marine *Streptomyces* sp. B9173. Genomics Appl Biol 34:1013–1020. (In Chinese.)

[4] Tatusova T, DiCuccio M, Badretdin A, Chetvernin V, Nawrocki EP, Zaslavsky L, Lomsadze A, Pruitt KD, Borodovsky M, Ostell J 2016 NCBI prokaryotic genome annotation pipeline. Nucleic Acids Res 44:6614–6624. doi:10.1093/nar/gkw569. 27342282 PMC5001611

[5] Borodovsky M, Lomsadze A 2014 Gene identification in prokaryotic genomes, phages, metagenomes, and EST sequences with GeneMarkS suite. Current protocol in microbiology. John Wiley & Sons. doi:10.1002/9780471729259.mc01e07s32. 24510847

[6] Camacho C, Coulouris G, Avagyan V, Ma N, Papadopoulos J, Bealer K, Madden TL 2009 Blast+: architecture and applications. BMC Bioinformatics 10:421. doi:10.1186/1471-2105-10-421. 20003500 PMC2803857

[7] Lowe TM, Eddy SR 1997 TRNAscan-SE: a program for improved detection of transfer RNA genes in genomic sequence. Nucleic Acids Res 25:955–964. doi:10.1093/nar/25.5.0955. 9023104 PMC146525

[8] Medema MH, Blin K, Cimermancic P, de Jager V, Zakrzewski P, Fischbach MA, Weber T, Takano E, Breitling R 2011 antiSMASH: rapid identification, annotation and analysis of secondary metabolite biosynthesis gene clusters in bacterial and fungal genome sequences. Nucleic Acids Res 39:W339–W346. doi:10.1093/nar/gkr466. 21672958 PMC3125804

[9] Zou Y, Fang Q, Yin H, Liang Z, Kong D, Bai L, Deng Z, Lin S 2013 Stereospecific biosynthesis of β-methyltryptophan from l-tryptophan features a stereochemical switch. Angew Chem Int Ed Engl 52:12951–12955. doi:10.1002/anie.201306255. 24166888

[10] Lan Y, Zou Y, Huang T, Wang X, Brock NL, Deng Z, Lin S 2016 Indole methylation protects diketopiperazine configuration in the maremycin biosynthetic pathway. Sci China Chem 59:1224–1228. doi:10.1007/s11426-016-0026-7.

[11] Xu F, Kong D, He X, Zhang Z, Han M, Xie X, Wang P, Cheng H, Tao M, Zhang L, Deng Z, Lin S 2013 Characterization of streptonigrin biosynthesis reveals a cryptic carboxyl methylation and an unusual oxidative cleavage of a N-C bond. J Am Chem Soc 135:1739–1748. doi:10.1021/ja3069243. 23301954

[12] Komaki H, Ichikawa N, Hosoyama A, Fujita N, Igarashi Y 2015 Draft genome sequence of *Streptomyces* sp. TP-A0890, a producer of FR-900452 and A-74863a. Genome Announc 3(5):e01212-15. doi:10.1128/genomeA.01212-15. 26472848 PMC4611700

[13] Wen Y, Wu X, Teng Y, Qian C, Zhan Z, Zhao Y, Li O 2011 Identification and analysis of the gene cluster involved in biosynthesis of paenibactin, a catecholate siderophore produced by *Paenibacillus* *elgii* B69. Environ Microbiol 13:2726–2737. doi:10.1111/j.1462-2920.2011.02542.x. 21883794

[14] Sandy M, Rui Z, Gallagher J, Zhang W 2012 Enzymatic synthesis of dilactone dcaffold of antimycins. ACS Chem Biol 7:1956–1961. doi:10.1021/cb300416w. 22971101

[15] Kodani S, Bicz J, Song L, Deeth RJ, Ohnishi-Kameyama M, Yoshida M, Ochi K, Challis GL 2013 Structure and biosynthesis of scabichelin, a novel tris-hydroxamate siderophore produced by the plant pathogen *Streptomyces* *scabies* 87.22. Org Biomol Chem 11:4686–4694. doi:10.1039/c3ob40536b. 23752895

